# The Distributed Practice Effect on Classroom Learning: A Meta-Analytic Review of Applied Research

**DOI:** 10.3390/bs15060771

**Published:** 2025-06-03

**Authors:** Rhys D. Mawson, Sean H. K. Kang

**Affiliations:** Faculty of Education, The University of Melbourne, Parkville, VIC 3010, Australia; rhys.mawson@hotmail.com

**Keywords:** distributed practice, spaced practice, spacing effect, meta-analysis, classroom learning

## Abstract

There is extensive evidence that distributed practice produces superior learning to massed practice, predominantly from laboratory studies often featuring decontextualized learning. A systematic review of applied research was undertaken to assess the impact of distributed practice on classroom learning. Inclusion criteria were classroom studies with learning materials and timescales relevant to curriculum-based learning. The screening of over 3000 articles resulted in 22 reports containing 31 effect sizes (N > 3000). A meta-analysis found a moderate effect in favor of distributed over massed practice (*d* = 0.54, 95% CI [0.31, 0.77]). Although a comprehensive quantitative moderator analysis was not possible due to the number of studies, generally larger effect sizes were associated with studies that featured longer retention intervals, had learners at higher education levels, and had fewer re-exposures to the materials.

## 1. Introduction

Although the trajectory of recent educational practices has moved away from the memorization of content (Pappas, 2009), repeated exposure to and the retention of foundational information is key to processing new information (Sweller, 2010). This remains true even in the context of 21st century classrooms aiming to promote 21st century skills (Saavedra & Opfer, 2012), where engagement with rich and complex tasks requires the scaffolded and timely retention of key knowledge (van Merrienboer et al., 2003). Thankfully, decades of research highlight several teaching and learning strategies that support and enhance learning and retention (Donoghue & Hattie, 2021; Dunlosky et al., 2013).

### 1.1. The Case for Distributed Practice

With a conceptualization refined over almost 150 years (Ebbinghaus, 1885/1913), distributed practice presents itself as a high-utility learning strategy (Dunlosky et al., 2013). Contrasted with *massed* practice, in which study material is presented in a back-to-back fashion, *distributed* or ‘spaced’ practice involves spacing out the same duration of study time across multiple study sessions (Rohrer & Pashler, 2010). Distributed practice involves the initial learning of content followed by one or more study sessions separated by a set time (i.e., the interstudy interval).

However, distributed practice is more than just learning spread out over time. Indeed, although some studies operationalize spaced practice as ‘stretched out’ study time (e.g., 26 h over 4.5 days versus 3 days; Breckwoldt et al., 2016), this misses the critical factor driving the benefits of spaced over massed practice. Specifically, theoretical explanations for the effect of distributed practice emphasize that it is the *repeated exposure to material* spaced over time that leads to efficient learning (Kang, 2016). For example, encoding variability theory suggests that information is likely to be encoded in different ways when repetitions are spaced (instead of massed), leading to multiple retrieval pathways and increased accessibility; according to the study-phase retrieval account, when an item is repeated after a gap in time, the learner is potentially reminded of its prior occurrence, prompting retrieval of the previous presentation, which enhances memory (Maddox, 2016). The deficient processing of massed repetitions is another theory—i.e., when information is repeated in quick succession, less attention is paid to the repetitions (Gerbier & Toppino, 2015). But this does not mean that more exposures automatically equal better retention. The nature of the gap between exposures also plays a role in the effect of distributed practice (Wiseheart et al., 2019). Indeed, evidence suggests that ‘expanding’ or increasingly long gaps between exposures have a positive effect on learning in some situations (Kang, 2016). In addition to spacing, the number of exposures is also important, with evidence that too many exposures can lead to fatigue that negatively impacts learning (Gurung & Burns, 2019). Although implementing the multiple exposures required to engage in ‘true’ distributed practice requires careful consideration, the consistently positive effect of distributed practice makes it a ‘desirable difficulty’ for both learners and educators (Bjork & Bjork, 2011).

Indeed, meta-analytic research emphasizes the benefit of distributed over massed practice on the retention of information (Cepeda et al., 2006; Donoghue & Hattie, 2021; Donovan & Radosevich, 1999; Janiszewski et al., 2003; Lee & Genovese, 1988). Moreover, the effect of this and other learning strategies are consistent across a range of characteristics of both the learners and learning involved, including age, learning domain, and ability level (Donoghue & Hattie, 2021). However, despite recent widespread research and communication regarding its positive impact (e.g., Dunlosky & Rawson, 2015; Kang, 2016; Rohrer & Pashler, 2010), distributed practice has a long history of being underutilized in educational settings (Dempster, 1988).

### 1.2. Bridging the Research–Practice Divide

The limited application of distributed practice likely stems from a disconnect between how learning is conceptualized in experimental settings and real-world classrooms (Ebersbach et al., 2022). Indeed, the ‘traditional’ laboratory-based study of distributed practice often involves very short retention (e.g., <10 min) and interstudy intervals (e.g., five seconds to one week) and is based predominantly on verbal learning tasks (see Cepeda et al., 2006; Janiszewski et al., 2003). Recent work to distill the relevance of this highly constrained research to educators does exist (Department of Education and Training, 2020; Pomerance et al., 2016). However, the highly specific evidence base for distributed practice means questions remain about the strategy’s application to real-world classrooms (Ebersbach et al., 2022).

Applied research into distributed practice has no doubt taken place, but its effect is often conflated with that of laboratory-based studies. For example, Donoghue and Hattie’s (2021) meta-analysis of learning strategies indicated a strong (*d* = 0.85) effect of distributed practice across studies including over 150,000 unique participants. While the authors reviewed the effect of this and other learning strategies across key variables, they did not distinguish between effect sizes drawn from classroom versus laboratory-based studies. As such, it is difficult to provide guidance on how to implement the strategy when there is no clear picture of the factors that influence its effect—positively and negatively—in everyday classrooms. As evidenced by similar research into other learning strategies (e.g., retrieval practice; Agarwal et al., 2021), a specific focus on classroom-based research could more clearly identify the effect of distributed practice in real-world settings.

Moreover, research conducted in educational settings more effectively captures the factors that influence real-world learning outcomes (Berliner, 2002). One of these contexts relevant to distributed practice is the various timescales of real-world learning. For example, it is increasingly common for school-based curriculum to require that students “progressively apply the familiar to develop new ideas” across year levels (Australian Curriculum Assessment and Reporting Authority, n.d.). In addition to this longer-term accumulation of knowledge, students also need to learn information in order to tackle upcoming classroom tasks and homework, show their knowledge at the end of units of work, or pass future exams (Foot-Seymour et al., 2019). Similarly, real-world classroom learning involves a range of materials and methods. In today’s classrooms, for example, students learn via approaches such as project-based learning (Lonergan et al., 2022), tackle 21st century skills like critical thinking (Foot-Seymour et al., 2019), and are often asked to go beyond surface-level learning (Hattie & Donoghue, 2016). Applied research into distributed practice helps bridge the research–practice gap by actively contending with the contexts of classroom learning.

Importantly, an up-to-date review would capture the recent resurgence of applied learning strategy research that is missing from previous reviews. Most notably, Donoghue and Hattie’s (2021) meta-analysis is based predominantly on the articles explored in Dunlosky et al.’s (2013) previous review of the learning strategy literature. Although comprehensive, the articles current at the time of Dunlosky et al.’s (2013) review are now more than 10 years old. Critically, there has been a growth of applied research on learning strategies in recent decades, likely a reflection of educators’ need to maximize limited classroom time (Agarwal et al., 2021; Kang, 2016). As such, the numerous articles published in the preceding 10 years likely add valuable information relevant to the application of distributed practice to classroom learning (e.g., Barzagar Nazari & Ebersbach, 2019a; Barzagar Nazari & Ebersbach, 2019b; Foot-Seymour et al., 2019; Greving & Richter, 2021; Lyle et al., 2019; Svihla et al., 2017). An updated review specifically focused on distributed practice research in applied settings is therefore both timely and feasible.

### 1.3. The Present Review

While the effect of distributed practice on learning is strong in laboratory-based studies, the effectiveness of this strategy on real-world classroom learning is less clear-cut. As such, a focus on applied research is required to paint a more accurate picture of the effectiveness of distributed practice on classroom learning. Moreover, a review focused on applied research would capture key aspects of classroom learning that may moderate the effect of distributed practice. These variables are critical to the successful and effective implementation of such a strategy.

The present review has two main objectives. First, this review aims to quantify the effect of distributed practice on primary, secondary, and university-level students’ ability to learn content relevant to their everyday classrooms. In addition, this review aims to explore the characteristics of classroom-based learning that may moderate the effect of distributed practice.

## 2. Method

PRISMA guidelines (see Page et al., 2021) were followed in this systematic review. Minor deviations (see, e.g., Section 2.6) are noted and explained in the sections outlined below.

### 2.1. Eligibility Criteria

Inclusion criteria were developed to focus this review on the application of distributed practice in mainstream educational settings. These criteria are summarized in Table 1 below. At the research level, the gray literature was not eligible for inclusion except for theses and dissertations. This concession was made because current information services systems mean theses and dissertations are more readily available forms of the literature than they have been previously (Schopfel & Rasuli, 2018). Moreover, doctoral dissertations in the field of educational psychology have a long history of being applied in focus (e.g., Ysseldyke & Pickholtz, 1975). Theses and dissertations therefore reflect an increasingly accessible source of information relevant to the aim of the present study.

In terms of study samples, the widespread emphasis on inclusive practices in education ensures that a wide range of learners—with and without disabilities—are participating and succeeding in mainstream classes (Anderson & Boyle, 2015). While research solely focused on special populations (e.g., Robinson, 1979) is important for a more nuanced understanding of the impact of this strategy and other learning strategies, it was excluded on the basis that it less effectively represents the broad types of classrooms that exist in mainstream learning environments.

For the intervention, a one-day interstudy interval was chosen as the smallest interval applicable to real-world learning. Specifically, everyday classrooms tend to cluster the learning of content, focusing on introducing new material in one class and then practicing it in the next lesson a day or so later (Svihla et al., 2017). A one-day interstudy interval is therefore relevant to the way that classrooms currently operate. Moreover, recent large-scale research suggests that optimal learning outcomes occur when the interstudy interval is approximately 10–20% of the required retention interval (Cepeda et al., 2008, 2009). While this indicates that a retention period of 7 days provides the most effective use of practice distributed over a day, retention periods both more and less than 7 days were included (i.e., no exclusions were placed on retention interval). This decision was motivated by the fact that the goal or purpose of learning defines the length of time needed to retain it, a factor which likely varies considerably across study levels, subject areas, and learning tasks.

Related to the study design criteria, the concept of ‘classroom-relevant learning materials’ was deliberately kept broad. This was due to the idea that relevance is defined by the learning environment, both in terms of the learners and learning domain. For example, pre-defining that the learning needed to be assessed formally (e.g., through exams) would have excluded primary school-aged learners whose learning is often more informally and qualitatively assessed. Similarly, a mathematics classroom in a primary school likely has access to very different resources and approaches to a graduate-level university course in accounting, in terms of how learning is presented, practiced, and assessed; methods for the distribution of practice and assessment of learning did not factor into exclusion and inclusion of studies, so long as they did not deviate substantially from what those learners might be expected to experience in their classroom. In general, studies were included if they took place in the classroom and involved content that would have been learnt by students in that class regardless of whether researchers had intervened.

### 2.2. Search Strategy

Articles were drawn from the Education Resources Information Centre (ERIC; ProQuest), PsycINFO (OVID), A+ Education (Informit), and ProQuest Central (ProQuest) databases. These databases were searched from their inception to June 2023. Common search terms were used for each database, including variants of *spaced*, *distributed*, *practice*, and *learning*. Changes were made to syntax as required by individual database guides. The search syntax used for each individual database is presented in Appendix A.

Citation searches were also used to supplement articles drawn from databases. Initially, reference lists included in recent meta-analyses and systematic reviews of the relevant literature were scanned for classroom-based studies meeting criteria (i.e., Donoghue & Hattie, 2021; Ebersbach et al., 2022). Following the initial screening and full-text review, a reference list search of the articles meeting inclusion criteria was also undertaken.

### 2.3. Selection Process

To monitor the inclusion and exclusion process, EndNote v20.6 (The EndNote Team, 2023) was used. One researcher (RM) reviewed the title and abstract of all studies to determine eligibility for full-text review. To monitor fidelity to the eligibility criteria, 10% of articles eligible for full-text review were also reviewed by the supervising researcher (SK).

To facilitate this, the citations for all full-text, non-duplicated reviews written in English (*n* = 194) were copied to Microsoft Excel (Microsoft Corporation, 2023). Excel’s ‘random number between’ function was used to generate 19 numbers between 2 and 195, corresponding with the row numbers of citations. The 19 associated titles, abstracts, and full-text articles were then independently reviewed by SK. Disagreements were resolved through discussion and consensus.

### 2.4. Data Items and Collection

The data extraction process was undertaken by one researcher (RM). This process involved the extraction of a range of data from the included literature, including study elements (e.g., year of publication), information about participants (e.g., education level), and key features of both the learning (e.g., learning domain or subject) and learning outcome (i.e., *M* and *SD* values for retention of information across conditions).

### 2.5. Synthesis Methods

To assess the effect of distributed versus massed practice, a Standardized Mean Difference (SMD) was calculated for every comparison in each study. Data relevant to mean retention at the longest retention interval were tabulated for the distributed and massed practice conditions in each study within a report. The mean retention of the massed condition was subtracted from that of the spaced condition and divided by a pooled standard deviation to create an effect size (i.e., Cohen’s *d*). Using this approach, positive values represent an effect in favor of distributed practice.

In all cases, reported means and standard deviations were used to calculate SMD values using an online effect size calculator (Wilson, n.d.). This process took place regardless of whether effect sizes were reported in individual studies, as researchers have a tendency to calculate effect sizes in multiple and sometimes conflicting ways (Lakens, 2013). This ensured consistency in the way that effect sizes were calculated. The random effect meta-analysis of the SMD values and their standard errors was conducted in R (v4.3.1; R Core Team, 2023) using the *metafor* package (v4.2-0; Viechtbauer, 2010). The decision to assume random effects was made given the significant differences in approaches to teaching and learning across countries, states, and individual schools. Output relevant to heterogeneity (e.g., *I*^2^ statistics) and its significance was also sought. The R code is included in the Supplementary Materials.

### 2.6. Bias and Certainty Assessment

Applied studies have an inherent focus on ecological validity. Hence, although high-quality applied experiments include elements of randomized-controlled trials, the two designs have distinct aims. As such, an explicit risk of bias assessment was deemed irrelevant. In terms of reporting bias, a funnel plot of effect sizes against standard errors for included studies was created for visual inspection. No certainty assessment was undertaken.

## 3. Results

### 3.1. Screening

**Figure 1:**
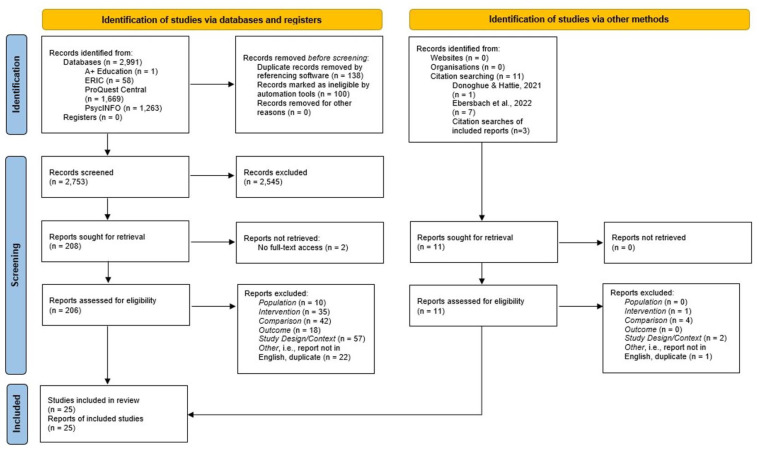
Flowchart of the screening process.

As highlighted in the flowchart (adapted from Page et al., 2021) in Figure 1 below, almost 90% of studies assigned to a full-text review were excluded. In addition to the application of the inclusion criteria above, studies were also excluded based on other features. For example, the greatest number of studies were excluded based on their study design. Here, studies were often decontextualized in comparison to real-world classroom learning, either because they were based in a lab (e.g., in a ‘simulated’ classroom; Kapler et al., 2015), used materials irrelevant to the class (e.g., primary school students studying vocabulary words from a postgraduate vocabulary reasoning exam; Sobel et al., 2011), or employed a combination of both (e.g., Suzuki & Dekeyser, 2017). In contrast, included studies involved learning tasks where the content was directly related to the specific course being studied (e.g., the statement of cash flows in an accounting course; McNellis, 2015) or was directly relevant to the general age and stage of the learner in more general classrooms (e.g., relevant spelling words for learners in a primary-school classroom; Petersen-Brown et al., 2023). In addition, a common theme was studies claiming to apply distributed practice when they were better described as examples of block scheduling. For example, several studies involving second-language word learning had students review groups of words either in one long session or across several shorter sessions (Namaziandost et al., 2019, 2020, 2023). However, these studies did not include repeated exposure to any one word. This represents a common misconception of distributed practice.

Another area of contention was the use of comparison groups that did not involve massed practice, made more challenging by the open definition of this condition in the inclusion criteria. For example, while some studies used easily excluded ‘business-as-usual’ conditions that involved neither massed nor distributed practice (e.g., Benson et al., 2022), others compared distributed practice to distributed ‘retrieval’. For example, Gjerde et al. (2022) had some students read through solutions to physics problems (review-only), while others were given problems to solve themselves (retrieval practice or ‘spaced testing’; see Agarwal et al., 2021). The lack of a massed practice comparison, and not the use of retrieval practice itself, led to the exclusion of this study. Similarly, distributed and massed practice conditions were included in Lindsey et al.’s (2014) study, but results from both were combined to compare against interleaved practice (for a definition and review, see Dunlosky et al., 2013). Overall, studies were excluded when distributed and massed practice conditions were not identifiable and clearly distinguishable.

### 3.2. Included Studies

#### 3.2.1. Included in Review

In total, 25 studies met all inclusion criteria. The full table of data extracted from the 25 studies is included under Supporting Materials.

#### 3.2.2. Included in Analysis

Despite 25 studies meeting criteria for review, not all studies were eligible for inclusion in a quantitative synthesis. For three reports (Ebersbach & Barzagar Nazari, 2020; Grote, 1995; and Barzagar Nazari & Ebersbach, 2018), outcome data could not be extracted or retrieved via contact with the authors. This resulted in 22 studies eligible for analysis.

Additional factors were also considered before conducting the quantitative analysis. This included the presence of unspecified or unequal retention periods, predominantly in which the timing between final exposure and the retention test was shorter for the distributed practice condition. In addition, non-independent effects existed in several included studies. While this was overcome in two studies by focusing only on results for retention tests and not those assessing students’ application of critical thinking skills (Foot-Seymour & Wiseheart, 2022; Foot-Seymour et al., 2019), non-independent effects remained in other studies. In those cases, additional standardized mean difference (SMD) values were created by combining the M and SD values across measures within each condition (i.e., massed and distributed) to create a single, independent effect. These combined values were calculated using the method outlined in the Cochrane Handbook for Systematic Reviews of Interventions (i.e., Chapter 6; Higgins et al., 2023) with the aid of an online tool (https://www.statstodo.com/CombineMeansSDs.php, accessed on 20 September 2023).

Effect sizes related to the above factors are clearly distinguished in the data extraction table (see Supplementary Materials). The potential impact of including or excluding those effects was explored through a preliminary analysis.

### 3.3. Synthesis

#### 3.3.1. Preliminary Analysis

Four preliminary meta-analyses were conducted (see Table 2 below). Each analysis employed a restricted Maximum Likelihood model under random-effect assumptions.

All analyses indicated a positive effect size, indicating overall results in favor of distributed over massed practice. In terms of differences, the qualitative comparison of Analyses 1 and 2 compared with 3 and 4 indicates that any overestimation due to unequal retention intervals in favor of the distributed practice condition is relatively small. In addition, replacing non-independent effect sizes with a single, average effect size for each relevant study resulted in a slight increase in the overall combined effect size (i.e., Analyses 2 and 4). Despite this, overall estimates based on the inclusion of non-independent effects (i.e., Analyses 1 and 3) are likely more accurate given that they are based on a greater number of effect sizes.

Acknowledging the above points, Analysis 1 appeared to maximize the amount of information for analysis while minimizing the inaccuracy introduced by averaging nonindependent effect sizes. As such, Analysis 1 is described in detail below.

#### 3.3.2. Main Analysis

**Figure 2:**
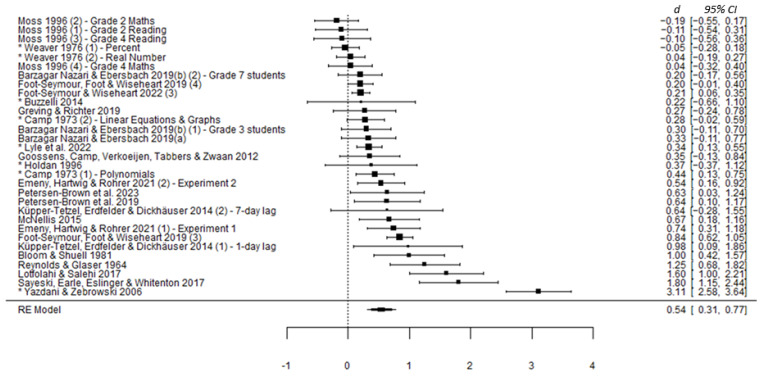
Forest plot of effect sizes included in final analysis. *Note*. An asterisk (*) denotes a study with an unequal/unspecified retention interval.

As confirmed visually in the forest plot in Figure 2 below, standardized mean difference (SMD) values across included studies showed an overall significant effect of distributed over massed practice (*d* = 0.54, 95% CI [0.31, 0.77], *z* = 4.631, *p* < 0.001). This indicates that the mean retention for learners in distributed practice conditions was, on average, over half a standard deviation higher than for those in massed practice conditions.

**Figure 3:**
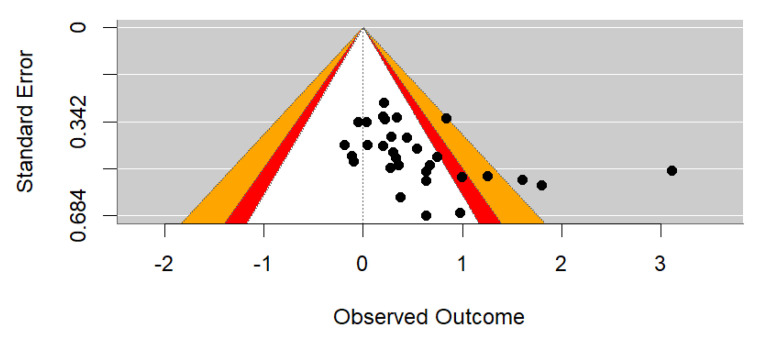
Contour-enhanced funnel plot of effect sizes from studies included in analysis (*k* = 22). *Note*. Orange = *p* < 0.01, red = *p* < 0.05, and white = *p* < 0.10 (i.e., statistically non-significant).

Of note, Egger’s test (*z* = 2.719, *p* = 0.007) confirms the significant asymmetry in the distribution of effects visible in Figure 3 below. However, as indicated by the highlighted areas of statistical significance, many studies were non-significant. This suggests that the asymmetry is unlikely due to publication bias. Instead, the asymmetry likely represents a ‘small study bias’, where methodological approaches inherent in small studies lead to stronger effects with greater variance (Peters et al., 2008; Sterne et al., 2011). The large (*d* = 3.11; *n* = 120) effect in the study by Yazdani and Zebrowski (2006) is a notable exception.

Notably, heterogeneity can also influence funnel plot asymmetry (Sterne et al., 2011). In the main analysis, the significant *I*^2^ value shown in Table 2 suggests that more than 92% of the variance between studies was due to systematic influence and not chance (*Q*(30) = 230.33, *p* < 0.001). A moderator analysis via meta-regression was considered as one method for exploring this systematic influence.

#### 3.3.3. Moderator Analysis

Several variables related to the timescales of and materials for learning were chosen a priori and extracted to facilitate a moderator analysis. However, guidelines recommend that 10 studies are required for every variable entered into the meta-analysis (i.e., two variables as appropriate for *k* = 22 studies); moreover, this is only true when there is an even distribution of covariates within a variable or characteristics (Deeks et al., 2023). Of the timescale variables, the data extraction table in the Supplementary Materials shows that none contained an appropriate amount of variability and/or were widely and accurately reported. For example, most studies used a fixed interstudy interval, while the amount of total study time was not reported in many studies. In addition, the retention interval could not be used as a moderator, given that several of the included studies made use of unequal or unspecified retention intervals. Of the learning material variables, ‘Learning Domain’ was the only factor widely reported and with good variability in levels.

When Analysis 1 was re-run with ‘Learning Domain’ as a moderating variable, results revealed a non-significant difference in the effect size at each level (*Q*(8) = 7.56, *p* = 0.478). This indicates that the estimated effect sizes within the nine learning domains were not significantly different from one another. This is evidenced by the overlap in estimated effect sizes within each learning domain, as shown in Table 3 below.

Moreover, the presence of significant levels of residual heterogeneity (*Q*(22) = 180.85, *p* < 0.001; *I*^2^ = 92.84%) indicates that this variable does not adequately explain the heterogeneity found in the main analysis.

The small amount of extracted data, as well as difficulties in extracting the required information from all studies, precluded additional moderator analyses. However, when grouped under key variables, qualitative differences in the size and direction of effect sizes were apparent. Effect sizes in the included literature were therefore grouped and reviewed based on relevant extracted variables (as per Agarwal et al., 2021). This approach is taken to illustrate possible trends in effects that, although beyond the scope of this study, require statistical exploration to understand whether *qualitative* differences are supported by *quantitative* analysis.

### 3.4. Key Variables

#### 3.4.1. Learning Domain

**Figure 4:**
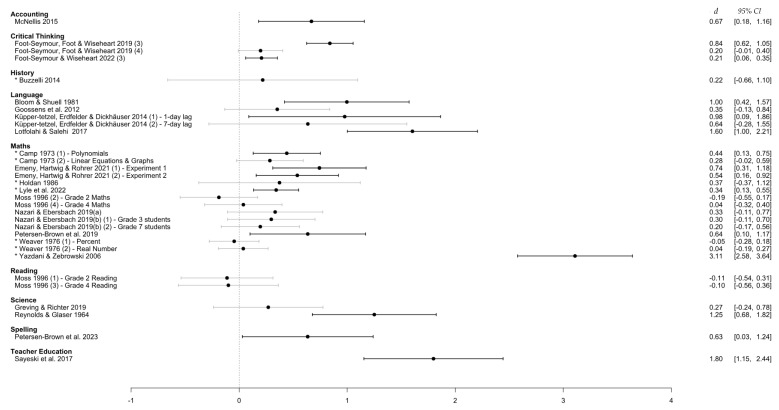
Effect sizes grouped by learning domain. *Note.* This figure shows the distribution of effect sizes when grouped by the area of learning (e.g., subject, course, or topic) that the content falls within. Grayed-out confidence intervals indicate those that cross the line of no difference (i.e., statistically non-significant). An asterisk (*) denotes a study with an unequal/unspecified retention interval.

As seen in Figure 4, consistently large effect sizes came from language learning studies. However, most effect sizes in this domain were drawn from classrooms focused on second-language learning. Indeed, only the single study by Goossens et al. (2012) suggested that the positive effect of distributed practice may generalize to vocabulary learning more generally. In comparison, the math learning domain had a larger number of studies, less variability in effects, and generally positive effect sizes for distributed over massed practice. Given that the included studies in this domain captured a range of math topics, distributed practice appears generally effective in the math domain.

#### 3.4.2. Retention Interval

**Figure 5:**
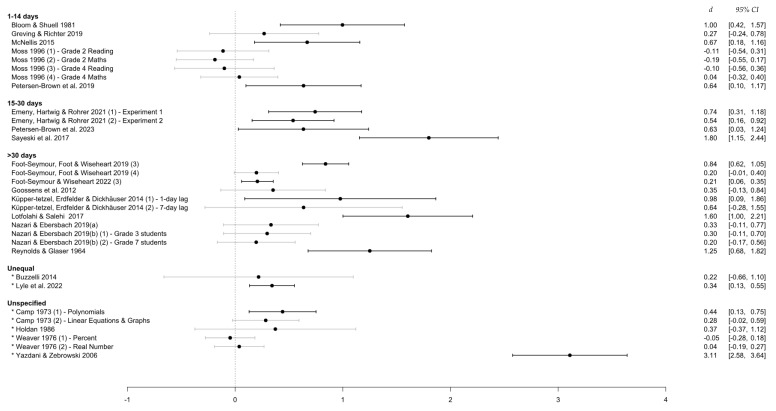
Effect sizes grouped by retention interval category. *Note.* This figure shows the distribution of effect sizes when grouped based on the length of time that participants have to retain the information, from the last study session to the assessment of learning outcomes. An asterisk (*) denotes a study with an unequal/unspecified retention interval.

Figure 5 highlights that the effect of distributed over massed practice tended to be larger at longer retention intervals. However, the present review did not find studies with intervals longer than 42 days, indicating a lack of information about the effects of distributed practice over very long-term scales. Of note, most studies with either unequal or unspecified retention intervals were still associated with positive albeit small effect sizes. However, the majority created conditions that favored those in the distributed practice condition, with final distributed practice sessions often taking place closer to the final test than the massed practice session. The fact that this ‘advantage’ was not associated with stronger effects in favor of distributed practice suggests the presence of other factors in these studies (see Section 4).

#### 3.4.3. Interstudy Interval

**Figure 6:**
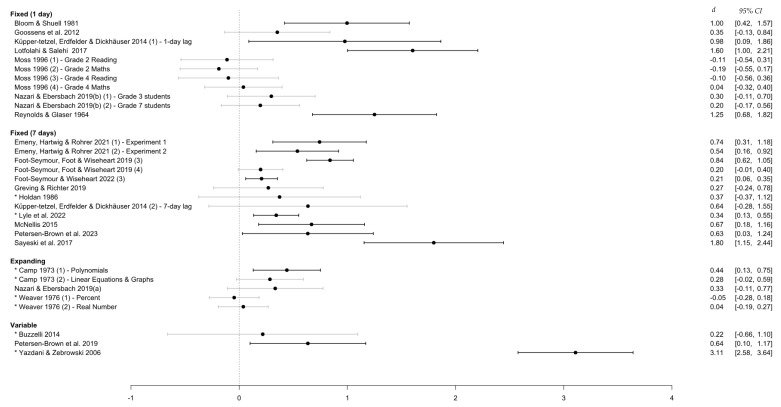
Effect sizes grouped by interstudy interval category. *Note.* This figure shows the distribution of effect sizes based on the length (1 versus 7 days) and nature (fixed versus expanding [i.e., increasing time between each re-exposure]) of the gap between study sessions. An asterisk (*) denotes a study with an unequal/unspecified retention interval.

Fixed intervals of seven days between re-exposures tended to produce the most consistently positive and significant effects for distributed over massed practice (see Figure 6). In addition, the effect sizes for expanding interstudy intervals tended to be smaller and, in one case, negative. However, the effect sizes for expanding intervals included here may not generalize, as most are based on the same study procedure.

#### 3.4.4. Education Level

**Figure 7:**
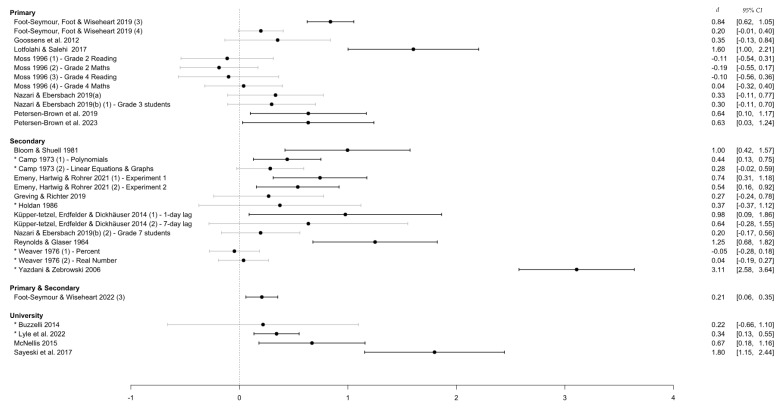
Effect sizes grouped by education level. *Note.* This figure shows the distribution of effect sizes based on whether participants are classified as primary-, secondary-, or university-level students by this study’s authors based on the cut-off ages and/or year levels used by the country in which this study took place. An asterisk (*) denotes a study with an unequal/unspecified retention interval.

Figure 7 shows that studies in primary school settings produced the largest number of negative effects (i.e., favoring massed over distributed practice). Moreover, studies at this education level had the highest proportion of non-significant effect sizes. In contrast, secondary school-level studies produced more consistently positive and significant effects. Interestingly, despite previous lab-based distributed practice research predominantly employing university-level participants, the present review captured only a small number of applied studies in university-level classrooms.

#### 3.4.5. Number of Re-Exposures

**Figure 8:**
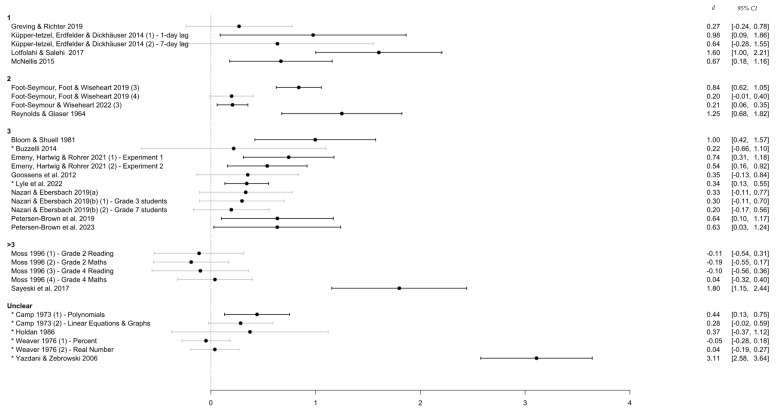
Effect sizes grouped by number of re-exposures. *Note.* This figure shows the distribution of effect sizes based on whether participants received 1, 2, 3, or more than three exposures to the content in the distributed practice condition. An asterisk (*) denotes a study with an unequal/unspecified retention interval.

Generally speaking, a smaller number of practice sessions resulted in a stronger effect in favor of distributed over massed practice. Indeed, Figure 8 shows that most studies with three exposures after the initial learning were associated with significant and moderate-to-large effect sizes. Of note, four of the five effect sizes drawn from distributed practice over more than three sessions came from one study. Indeed, the number of re-exposures used in Moss (1996) was considerably larger than much of the included literature (i.e., nine re-exposures to each of the reading and math learning material). While it was not possible to ascertain the exact number of re-exposures due to a lack of reporting, most of the studies in the ‘unclear’ level of this variable also described a procedure that included a high number of exposures (i.e., up to 5). Given that these effect sizes were also smaller than those of studies with fewer exposures, it is likely that these studies provide further evidence for the trend of ‘less is more’ when it comes to practice sessions.

### 3.5. Key Themes

In addition to the above variables, a broader look at the included literature revealed several key themes. These themes represent areas of the design of the included studies, which may provide helpful information for implementing distributed practice in real-world classrooms.

#### 3.5.1. The ‘Practice’ in Distributed Practice

While all studies involved practice via repeated exposure to the learning material, the form of that ‘exposure’ differed greatly across the literature. For example, in studies utilizing distributed practice for language or vocabulary learning (e.g., Bloom & Shuell, 1981), repeated exposure was simply a re-presentation of the same words. In contrast, the studies involving math learning tended to present practice problems that, although based on the same content, were not the exact same problems with the exact same numbers. The study by Lyle et al. (2022), for example, described the different practice problems as “algorithmic variants” (p. 1803). Although repeatedly exposed to the same general idea, learners in this and similar studies rarely—if ever—saw the exact same thing in each exposure. In real-world classrooms, the nature of repeated exposure appears to depend on the content being learnt.

The *process* of these repeated exposures also differed across studies. For example, some studies focused solely on repeated exposure (e.g., re-reading the same passage; Greving & Richter, 2019), others involved learners working on problems associated with the initially learnt content (e.g., practice math problems; Weaver, 1976), while others still asked learners to review and practice content through the use of complex exercises (e.g., the Cover–Copy–Compare process for learning spelling words; Petersen-Brown et al., 2023). Moreover, the studies aiming to improve students’ critical thinking (i.e., Foot-Seymour et al., 2019; Foot-Seymour & Wiseheart, 2022) had the additional complexity of explicitly asking students to transfer or apply information to completely new situations. These variations on the process of practice are all highly relevant to the learning process in real-world classrooms.

#### 3.5.2. Feedback in Practice and the Role of Technology

Moreover, the nature of how the learning material was practiced also influenced the extent to which learners could receive feedback on their learning across exposures. In particular, the provision of feedback depended on the extent to which repeated exposures asked students to remember or simply review the information. For example, Buzzelli’s (2014) use of tweets via Twitter focused on giving students the information presented in earlier lectures in a different, additional form. As such, each ‘practice’ session or exposure did not require action from learners other than reading. In contrast, the study by Petersen-Brown et al. (2019) involved learners giving definitions for exposed words, and, when they provided incorrect definitions, they were given feedback and then asked to correctly recall the definition again later. As such, these learners also received more exposure than those who correctly recalled the definitions initially. Learning to criterion (i.e., where an item correctly recalled a set number of times is removed from further review) has been criticized as an unnecessary confounding factor in distributed practice research (Cepeda et al., 2006); it is one that appears to have made its way into applied studies. In general, feedback was more common in studies that involved active engagement from learners rather than just passive review.

In addition, the ability for learners to receive feedback was heavily influenced by whether technology formed a part of the practice process. For example, while practice problems were a common theme in studies of math learning, these problems were presented in different ways. The use of an online math-practice platform in the study by Lyle et al. (2022) enabled learners to receive feedback on practice problems after completing the relevant quizzes. In contrast, participants in studies by Camp (1973) and Holdan (1986) did not receive feedback on completed textbook questions. In studies involving non-math learning, the use of digital flashcards in Sayeski et al.’s (2017) study also enabled real-time feedback. Moreover, the feedback was presented by a peer, something also employed in other included studies but without technology (i.e., Lotfolahi & Salehi, 2017). Where the feedback comes from adds further complexity to the question of how feedback influences the success of distributed practice specifically and learning more generally.

The provision of feedback across distributed practice sessions also highlights the interrelationship between spaced *practice* and spaced *testing*. This has been highlighted previously as a widely used strategy in today’s classrooms (Agarwal et al., 2021). Given that the combination of these two related approaches to repeated exposure can influence learning (Gurung & Burns, 2019), clearly distinguishing between the effects of these two strategies is key to the success of their respective implementations in classrooms. Indeed, a key differentiating factor may be the extent to which the repeated exposure is facilitated by the teacher (i.e., testing) or the learner themselves (i.e., studying).

#### 3.5.3. Distributed Practice as a Teaching or Learning Strategy?

The literature varied widely in the extent to which distributed practice was viewed as a teaching versus learning strategy. None of the included studies gave learners the option to practice materials in a distributed fashion without requiring it, a necessary consequence of experimental studies in which procedures are pre-determined and adherence is expected. Instead, studies differed in the extent that teachers drove the practice sessions. For example, approaches included sessions in which learners practiced on their own (e.g., Camp, 1973), those in which peers practiced with each other without a teacher (e.g., Lotfolahi & Salehi, 2017; Sayeski et al., 2017), studies involving teachers working alongside individual students (e.g., Petersen-Brown et al., 2019), and those in which teachers directly ‘taught’ the content in each exposure (e.g., McNellis, 2015). However, the positive effects across this range of implementation approaches likely reflect the versatility of distributed practice in real-world classrooms.

## 4. Discussion

### 4.1. The Overall Effect

Compared to massed practice, distributed practice had a moderate effect (*d* = 0.54) on classroom-based learning of curriculum-relevant materials. This provides clear evidence in favor of using distributed practice to support everyday classroom learning. However, results of the present study indicate a somewhat smaller overall effect in comparison to previous reviews (*d* = 0.85; Donoghue & Hattie, 2021). The fact that the Donoghue and Hattie (2021) meta-analysis combined the effects of distributed practice in experimental and applied settings is a likely factor in this difference.

Differences in effect sizes across such designs have previously been attributed to differences in the complexity of the learning that takes place in them (Ebersbach et al., 2022). Indeed, previous meta-analyses of distributed practice highlight that its effect is often stronger for less complex tasks, where complexity is based on the requirements of and decisions to be made within the task (Donovan & Radosevich, 1999). This fits with the findings of the present review.

Specifically, a key component of complexity in classroom learning is what learners need to do with the knowledge gained. While the decision was made in the present review to focus solely on the retention of information, several included studies employed learning and practice that went beyond just restating previous information. For example, most studies in the included literature involved math learning and used distributed practice conditions in which learners completed practice questions. However, each question was not simply a restatement of previous questions and tended to include slight variations, such as different values or contexts. As effect sizes drawn from math-learning studies tended to be smaller and more frequently non-significant in the present review, it is possible that the added complexity of needing to transfer learning to relatively novel situations meant that such studies captured something more complex than ‘surface level’ learning (Hattie & Donoghue, 2016). The availability of clear and easily applied definitions for levels of learning is therefore important to future reviews of applied distributed practice research.

However, the complexity of materials is unlikely to be the only factor relevant for educators seeking to effectively implement distributed practice. Indeed, a qualitative synthesis of the included studies highlighted differences in effect sizes across a range key variables.

### 4.2. Timing as a Factor

As expected, based on previous laboratory-based research, the present study indicated that the interstudy interval remains a key factor in the size of the effect of distributed practice over massed practice. For example, studies with a 7-day interstudy interval were consistently statistically significant and often associated with moderate-to-large effect sizes when compared to massed practice. However, in real-world classrooms, most teaching and learning tend to be condensed into distinct units that are covered intensely over a short number of days, rather than being covered in a single longer session as is done in massed practice conditions (Svihla et al., 2017). To better understand the effect of timing on distributed practice, future research could use alternative comparison conditions (e.g., 1-day interstudy intervals) that align more closely to classroom learning.

Similarly, the higher frequency of larger effects for studies with longer intervals found in this study broadly reflects the pattern seen in laboratory studies. However, such research also emphasizes that the effect of retention intervals on the distributed practice advantage does not continuously increase as the retention interval increases (Cepeda et al., 2008). Also, there is evidence from lab experiments with just one re-exposure that the optimal interstudy interval depends on the retention interval (Cepeda et al., 2009). However, the relationship between interstudy and retention intervals was not something that could be assessed with the included data, given the somewhat restricted nature of retention intervals employed (i.e., <42 days) and the varying numbers of re-exposures. Ongoing research in applied settings would benefit from a focus on this relationship.

Finally, studies with unequal or unspecified retention intervals require individual consideration. While retention interval decisions in these studies were somewhat constrained by efforts to fit the experiment into the existing school calendar, they nevertheless created conditions that appeared to favor distributed practice conditions (i.e., shorter retention intervals). Despite this apparent advantage, studies with unequal retention intervals were associated with smaller effect sizes, even compared to those with which they shared other methodological aspects (e.g., same learning domain). Influential factors here may have been the common use of expanding interstudy intervals or the higher number of re-exposures used in these studies. The negative impact of these factors fits with evidence that, in scheduling too many exposures to content, learning can be negatively impacted through increasing students’ fatigue from constantly reviewing the content (Gurung & Burns, 2019).

While highlighting the complexities of both planning for and implementing effectively timed distributed practice, the present study nevertheless captures a range of time scales more relevant to real-world classrooms. Critically, results suggest that distributed practice remains broadly effective across this range.

### 4.3. Learning Domain and Level

The magnitude—but not direction—of effect sizes favoring distributed practice appears to differ across learning domain (i.e., subject area). However, unlike previous reviews (i.e., Donoghue & Hattie, 2021), the meta-regression in the present did not confirm the statistical significance of this apparent difference. This may have been caused by differences in the variability of learning domains captured in the included studies relative to previous research. For example, studies involving learning in English or Reading made up 35.1% of the included studies in Donoghue and Hattie’s (2021) review but only 6% in the present review. The larger proportion in the former may reflect that the included laboratory-based studies involving language-based learning (e.g., verbal paired associates) were considered most closely aligned to those learning domains. Generally speaking, areas beyond language and math learning appear underrepresented in the applied literature, and, except for the studies applying distributed practice to critical thinking (Foot-Seymour et al., 2019; Foot-Seymour & Wiseheart, 2022), distributed practice is yet to be widely applied to 21st-century skills learning. These represent critical areas for future study.

In terms of education level, studies set in secondary schools were more often associated with stronger positive effects of distributed practice on learning than those in primary school settings. Although smaller in magnitude, this difference in effects is reflected in the spread of data in previous meta-analyses, where the effect of distributed practice was generally smaller for primary school learners (mean Cohen’s *d* = 0.57) than for those in secondary schools (mean Cohen’s *d* = 0.70; Donoghue & Hattie, 2021). However, although viewing effect sizes through the lens of educational levels confirms stronger positive effects for older children, descriptive statistics for age were not widely reported.

As such, the present review could not untangle whether positive effects are due to some element of learning environments involving older learners or a variable associated with age more generally. For example, most secondary schools tend to compartmentalize learning into separate units within isolated subjects, albeit this is a trend that more progressive learning approaches such as project-based learning attempt to reshape (Lonergan et al., 2022). It is therefore possible that, in using more cross-curricular approaches to learning, primary students engage in more complex learning that is less amenable to distributed practice. Similarly, the nature of how classes are timetabled at secondary schools versus primary schools differs significantly, particularly the way in which secondary schools focus on more discreetly and inflexibly blocked learning times or how primary schools tend to keep the same groups of students together across classes. These aspects of classrooms at each education level could impact both the study of distributed practice in applied research and the effects found within. Isolating the effects of age from educational level, as well as a closer look at the nature of the learning environment at each level, is likely to be a fruitful avenue for future research.

### 4.4. Limitations

Although providing evidence for the application of distributed practice in everyday classrooms, the present review has some limitations. For example, despite the decision to use narrow search criteria, our preliminary analysis indicated significant levels of heterogeneity in the included literature. This may have been driven by the fact that some elements of the inclusion criteria were not well defined. Specifically, distributed practice was defined based on timing rather than on the process and content of the practice itself. As discussed in the Results Section, this led to comparisons between disparate studies that varied in terms of how content was re-exposed and what learners needed to do with it. Further refinement of a definition for the ‘practice’ in distributed practice may decrease heterogeneity in future reviews.

In addition, the methods for both the calculation and interpretation of effect sizes in this study may have influenced our results. Indeed, the methods used to create effect sizes may have introduced inaccuracy into the measurement of the distributed practice effect. Specifically, the calculation of standardized mean effect sizes was conducted using extractable data, rather than relying on the effect sizes presented in individual studies. While this decision was made to avoid the various ways that effect sizes are calculated in the literature generally (Lakens, 2013) and in learning strategy research specifically (see Agarwal et al., 2021), such an approach may mean that the critical information influencing each study author’s statistical decision making was lost. Also, the interpretation of effect sizes across variables in this study was based on *qualitative* rather than *quantitative* analysis. This decision was heavily influenced by the lack of statistical power in the collated data. While this study provides an important initial step in understanding differences in effects within variables, larger sample sizes are required for a formal moderator analysis.

In a handful of the studies that focused on mathematics, practice assignments in the distributed practice condition—but not the massed practice condition—featured a mixture of different topics (Camp, 1973; Holdan, 1986; Weaver, 1976; possibly Yazdani & Zebrowski, 2006). In other words, in a few cases, distributed practice was conflated with interleaved practice, which itself has been shown to benefit mathematics problem solving (e.g., Taylor & Rohrer, 2010), and, thus, it is unclear to what extent the effects observed in these studies are due to spacing vs. interleaving. Having said that, it is worth noting that there is evidence that the interleaved practice effect in mathematics is caused by distributed practice (Foster et al., 2019).

Finally, the present study is unable to make specific comments on the effect of such a strategy on special populations (e.g., autistic learners). It can be assumed that students with learning and developmental disorders would be included in representative samples of inclusive classrooms. However, data relevant to sample representativeness were not reported, and, hence, the assumption cannot be guaranteed. Also, effect sizes are calculated from group averages and do not tell us whether or which subset(s) of the sample may have benefited to a different degree from distributed practice. Future research could consider participant samples more closely or focus on special populations specifically.

### 4.5. Implications

#### 4.5.1. Educational Implications

The generally positive effect of distributed practice—across a wide range of approaches to that practice—provides clear evidence that re-exposure to material in any form across separate sessions is likely to improve learning. While results of the present study highlight the benefit of this strategy for math learning and in secondary school settings in particular, findings more generally suggest that distributed practice can—and should—be broadly applied. As such, educators across curriculum areas and within schools that use a range of teaching methods are encouraged to explore their use.

Importantly, this research emphasizes that the interplay between timing variables, as well as their respective and combined influence on the effect of distributed practice, represents a complex set of considerations for educators. Indeed, one of the challenges in the included literature was the impact of needing to fit the distributed practice around pre-defined tasks such as end-of-unit assessments or semester exams. Far from discouraging the application of distributed practice in real-world classrooms, the present study highlights the importance of embedding distributed practice during the planning phase of creating individual units and broader subjects. In particular, teachers are encouraged to think about when the learning will be used (e.g., for a test at the end of a four-week unit) to help them make decisions about spacing out the associated practice.

#### 4.5.2. Research Implications

Despite being a clearly stated aim of the present study, the quality of the available research—in both its focus and reporting—hampered the effort to create practical guidance for educators looking to implement this strategy. For one, the present study illustrates a disconcerting level of unclearly reported or missing data across key variables. This impacts the capacity of researchers to offer defensible implementation suggestions even on variables widely considered to impact the effect of this strategy. Alongside more consistent reporting of those variables, it is critical that researchers actively question whether these are the variables of most interest to educators. For example, the present study provides preliminary—but quantitatively unsubstantiated—information on what elements of learning are being distributed and how, as well as the role that technology and feedback play in its spacing and retrieval. A widespread focus on these factors will enable researchers to provide advice to educators that is more relevant and easily implemented, across curriculum areas and levels of study.

## 5. Conclusions

Distributed practice plays a positive and significant role in classroom learning outcomes. Undoubtedly, its implementation requires consideration of the nature of the learning and the timescales over which it needs to take place. Nevertheless, embedding distributed practice into the planning of units and subjects enables teachers and students across educational levels and curriculum areas to reap the benefits of this strategy.

## Figures and Tables

**Table 1:** Summary of eligibility criteria for the systematic review.

	Inclusion	Exclusion
Population	Study participants enrolled in primary, secondary, or tertiary education	Participants drawn exclusively from special populations (e.g., autistic, intellectual disability, etc.)
Intervention	Distributed or spaced practice with interstudy intervals of at least 1 day	Interstudy intervals of less than 1 day
Comparison	Massed practice conditions with time spent studying equal to that of spaced practice conditions	Comparison to other learning strategies
Outcomes	Measures of retention (e.g., percentage correctly recalled) or transfer of information learnt (e.g., percentage correctly applied). Where retention was measured more than once, data were extracted from the longest retention interval measured in both the comparison and intervention conditions	Qualitative outcomes (e.g., teacher and/or student perceptions of effectiveness)
Study design and context	Studies conducted in the classroom using classroom-relevant learning materials	Studies using learning materials with no clear connections to specific syllabi or curriculum

**Table 2:** Summary of meta-analyses conducted.

Analysis	Potential Data Inaccuracies		Studies	Effect Sizes	Effect Size [95% CI]	*I*^2^ (%)
	Unequal Retention Intervals	Non-Independent Effect Sizes				
1	Included	Included All	22	31	0.54 [0.31, 0.77]	92.13
2	Included	Averaged	22	25	0.63 [0.36, 0.89]	94.93
3	Excluded	Included All	16	23	0.51 [0.31, 0.72]	83.88
4	Excluded	Averaged	16	19	0.57 [0.36, 0.78]	84.68

**Table 3:** Results of the random-effect meta-regression with ‘Learning Domain’ as a moderator.

Learning Domain	Effect Size [95% CI]
Accounting	0.67 [0.18, 1.16]
Critical Thinking	0.41 [0.00, 0.82]
History	0.22 [0.00, 0.44]
Language	0.91 [0.44, 1.3]
Math	0.46 [0.09, 0.84]
Reading	−0.11 [−0.42, 0.21]
Science	0.75 [−0.21, 1.71]
Spelling	0.63 [0.03, 1.24]
Teacher Education	1.80 [1.15, 2.44]

*Note.* Meta-regression model uses the ‘Accounting’ level of the variable as the reference level.

## Data Availability

Data supporting reported results will be made available by the authors upon request.

## Supplementary Materials

The following supporting information can be downloaded at https://www.mdpi.com/article/10.3390/bs15060771/s1: full tables of extracted data and R code. References (Emeny et al., 2021; Küpper-tetzel et al., 2014; Reynolds & Glaser, 1964) are cited in the supplementary materials.

## Appendix A

**Table A1:** Search syntax by database.

Database	Search Syntax	Search Fields
A+ Education (Informit)	(“spacing effect” OR “distribut* practi?e”~1 OR “spac* practi?e”~1 OR “mass* practi?e”~1 OR “distribut* learn*”~1 OR “spac* learn*”~1 OR “mass* learn*”~1) AND (class* OR stud* OR learn OR appli*)	Default
ERIC	(“spacing effect” OR “(distribut* OR spac* OR mass*) N/1 practi#e” OR “(distribut* OR spac* OR mass*) N/1 learn*”) AND (class* OR stud* OR appli*)	Default (All Fields+ text)
PsycINFO (Ovid)	((spacing effect or ((distribut* or spac* or mass*) adj practi#e) or ((distribut* or spac* or mass*) adj learn*)) and (class* or stud* or appli*))	Default (Title, Abstract, Heading Word, Table of Contents, Key Concepts, Original Title, Tests & Measures, Mesh Word)
ProQuest Central (ProQuest)	(“spacing effect” OR “(distribut* OR spac* OR mass*) N/1 practi#e” OR “(distribut* OR spac* OR mass*) N/1 learn*”) AND (class* OR stud* OR appli*)	Default (All Fields+ text)
