# Supplementary material for: The Distributed Practice Effect on Classroom Learning: A Meta-Analytic Review of Applied Research
Source: Behav Sci (Basel). 2025 Jun 3;15(6):771. doi: 10.3390/bs15060771 (PMC12189222; doi:10.3390/bs15060771)
Supplement: Supplementary file 1 [file behavsci-15-00771-s001.zip › behavsci-3588393-supplementary.pdf]

# Full Table of Extracted Data

| Author | Year | Country of first author | Report Type | Design | *N* | Age M(SD) | Enrolled Education Level | Massed Practice (MP) | Distributed Practice (DP) | Total time spent studying (mins.) | Re-exposures | DP Interstudy interval | Longest Retention interval (days) | Learning Domain | Learning Lead | Measure | Massed M (SD) | Massed *n* | Distributed M (SD) | Distributed *n* | Cohen's *d* | 95% CI Lower | 95% CI Upper | SE |
|---|---|---|---|---|---|---|---|---|---|---|---|---|---|---|---|---|---|---|---|---|---|---|---|---|
| | Study | | | | | Population | | Study Conditions | | | Timing | | | Learning | | Outcome | | | | | | | | |
| Bloom & Shuell | 1981 | USA | Unpublished Dissertation | Between | 52 | Not report (US Grades 9-12) | Secondary | 3 10-minute exercises (Multiple choice quiz, 'fill in the gaps' sentences, practice test), undertaken consecutively | Same as MP but spaced 1 day apart | Equal (30 minutes) | 3 | Fixed (1 day) | 3 | Language | Researcher | Retention - Number of words correct (out of 20) | 11.15 (4.02) | 26 | 15.04 (3.78) | 26 | 1.00 | 0.42 | 1.57 | 0.29 |
| * Buzzelli | 2014 | USA | Unpublished Dissertation | Between | 20 | Not reported | University | Review sheet containing similar content to the DP tweets, provided at the end of the 4-week unit (4 days before exam) | 6 tweets (3 straight after learning the concept, 3 more 3-5 days after initial learning) for each of the 15 concepts, spaced across the 4-week unit | Not reported | 3 | Variable (3-5 days) | Unequal | History | Teacher | Retention - Number of multiple choice questions correct (out of 15) | 12.00 (1.41) | 10 | 12.30 (1.34) | 10 | 0.22 | 0.66 | 1.10 | 0.11 |
| * Camp | 1973 (1) - Polynomials | USA | Unpublished Dissertation | Between | 163 | Not report (US Grades 8-9) | Secondary | A set number of homework problems were set for the class after the initial learning | The number of homework problems given to the massed practice condition were distributed based on a pre-determined mathematical function that provided the number of problems at day 1 (the day the topic was introduced) and then at days 2, 7, 12, 21 and 38 as required to complete all problems | Not reported | Unclear | Expanding (1,2,4,7,15,21, 38) | Unspecified | Maths | Teacher | Retention - Polynomial Chapter Quiz (out of 15) | 6.79 (3.61) | 81 | 8.42 (3.78) | 82 | 0.44 | 0.13 | 0.75 | 0.16 |
| * Camp | 1973 (2) - Linear Equations & Graphs | | | | | | | | | | Unclear | | | | | Retention - Linear Equations and Graphs chapter quiz (out of 14) | 4.76 (2.53) | 81 | 5.49 (2.60) | 82 | 0.28 | -0.02 | 0.59 | 0.16 |
| * ^ Camp | 1973 (3) - Average (across content) | | | | | | | | | | Unclear | | | | | Retention - across both quizzes | 5.775 (3.27) | 162 | 6.955 (3.5523) | 164 | 0.35 | 0.13 | 0.56 | 0.11 |
| Ebersbach & Barzagar Nazari | 2020 | Germany | Published Empirical Study | Between | 105 | MP - 23.2 (2.6) DP - 42.1 (4.2) | University | Crammed' condition, 12 practice items (same content, different contexts and numbers) given to participants 7 days after final lecture | 3 problem sets of 4 items each, provided at 0 (straight after last lecture), 2 and 5 days after the final lecture) | Not reported | 3 | Expanding (0, 2 and then 5 days) | 35 | Maths | Teacher | Retention - Percentage of total attainable score based on four test items | Not reported | 64 | Not reported | 41 | 0.22 | NA | NA | NA |
| | | | | | | | | | | | | | | | | Transfer - percentage of total attainable score based on 9 items (T/F) assessing general understanding | Not reported | 64 | Not reported | 41 | 0.43 | NA | NA | NA |
| Emeny, Hartwig & Rohrer | 2021 (1) - Experiment 1 (significant differences in prior maths knowledge) | UK | Published Empirical Study | Within | 44 | Not reported (UK Year 7) | Secondary | 12 problems provided immediately after a audiovisual tutorial | Same 12 problems, with 4 presented immediately after the tutorial, 4 presented 1 week later and the final 4 1 week after that. | Equal (25 minutes, including task completion, feedback and error correction for each item) | 3 | Fixed (7 days) | 28 | Maths | Teacher | Retention - Percentage of total attainable score based on four novel problems | 27.8 (32.4) | 44 | 55.1 (40.6) | 44 | 0.74 | 0.31 | 1.18 | 0.22 |
| Emeny, Hartwig & Rohrer | 2021 (2) - Experiment 2 (no significant difference in prior maths knowledge) | | | | 55 | | | | | | 3 | | | | | | 38.2 (37.5) | 55 | 59.1 (40.1) | 55 | 0.54 | 0.16 | 0.92 | 0.19 |
| Foot-Seymour, Foot & Wiseheart | 2019 (1) - Critical Thinking (application of 4 categories for | Canada | Published Empirical Study | Between | 387 | Not reported (Canadian Grades 4-6) | Primary | Initial learning session on how to evaluate website credibility before evaluating two websites, across 2 | Same website evaluation learning session followed by one lesson each week for the next 2 weeks (i.e., 2 weeks total) | Equal (approx. 270 minutes) | 2 | Fixed (7 days) | 35 | Critical Thinking | Teacher as Researcher | Transfer - mean percentage of categories used in explanation of website credibility | 17.2 (21.6) | 210 | 28.8 (26.6) | 177 | 0.48 | 0.28 | 0.69 | 0.10 |

| | | | | | | | | | | | | | | | | | | | | | | | | |
|---|---|---|---|---|---|---|---|---|---|---|---|---|---|---|---|---|---|---|---|---|---|---|---|---|
| | evidence evaluation) | | | | | | | lessons 'massed' over the next two days (i.e., 3 days total) | | | | | | | | | | | | | | | | |
| Foot-Seymour, Foot & Wiseheart | 2019 (2) - Critical Thinking (application of 17 questions for evaluating credibility) | | | | | | | | | | 2 | | | | | Transfer - mean percentage of key questions used in explanation of website credibility | 18.6 (11.0) | 210 | 24.2 (12.1) | 177 | 0.49 | 0.28 | 0.69 | 0.10 |
| Foot-Seymour, Foot & Wiseheart | 2019 (3) - Fact learning (categories recalled) | | | | | | | | | | 2 | | | | | Retention - mean percentage of categories remembered in cued recall test | 30.8 (28.8) | 186 | 56.2 (31.7) | 176 | 0.84 | 0.62 | 1.05 | 0.11 |
| Foot-Seymour, Foot & Wiseheart | 2019 (4) - Fact learning (questions recalled) | | | | 362 | | | | | | 2 | | | | | Retention - mean percentage of questions recognised amongst 'lure' questions | 62.6 (31.1) | 186 | 68.4 (27.2) | 177 | 0.20 | -0.01 | 0.40 | 0.11 |
| ^ Foot-Seymour, Foot & Wiseheart | 2019 (5) - Average (across recall scores in (3) and (4)) | | | | | | | | | | 2 | | | | | Retention - combined mean of (3) and (4) | 46.7 (33.9027) | 372 | 62.3173 (30.1135) | 353 | 0.49 | 0.34 | 0.63 | 0.08 |
| Foot-Seymour & Wiseheart | 2022(a) - Critical Thinking (evaluation categories applied to justifying website credibility) | | | | | | | | | | 2 | | | | | Transfer - mean number of categories used in explanation of website credibility (combined across two websites, out of 4) | 2.2351 (1.0979) | 728 | 2.1506 (1.1659) | 688 | -0.07 | -0.18 | 0.03 | 0.05 |
| Foot-Seymour & Wiseheart | 2022(b) - Critical thinking (evaluation questions applied to justifying website credibility) | Canada | Published Empirical Study | Between | 716 | MP - 12.0 (1.1) DP - 11.8 (1.1) | Primary & Secondary | Same as Foot-Seymour, Foot & Wiseheart (2019), although initial lesson was pre-recorded by researcher while other lessons were facilitated by teacher and used online materials | Same as Foot-Seymour, Foot & Wiseheart (2019) and as per MP | Equal (appox. 300 minutes) | 2 | Fixed (7 days) | 35 | Critical Thinking | Teacher | Transfer - mean number of key questions used in explanation of website credibility (combined across two websites, out of 17) | 3.9414 (2.4199) | 728 | 3.921 (2.5584) | 688 | -0.01 | -0.11 | 0.10 | 0.05 |
| Foot-Seymour & Wiseheart | 2022(c) - Fact learning (cued recall of website evaluation categories) | | | | | | | | | | 2 | | | | | Retention - mean number of categories remembered in cued recall test (out of 4) | 2.3 (1.4) | 367 | 2.58 (1.3) | 349 | 0.21 | 0.06 | 0.35 | 0.07 |

| | | | | | | | | | | | | | | | | | | | | | | | | |
|---|---|---|---|---|---|---|---|---|---|---|---|---|---|---|---|---|---|---|---|---|---|---|---|---|
| Goossens, Camp, Verkoeijen, Tabbers & Zwaan | 2012 | Netherlands | Published Empirical Study | Within | 33 | All - 8.91 (0.40) | Primary | Students learnt 30 words from Grade 4 learning material in the initial session, from which two lists of 15 words were created. One list was learnt in a MP fashion, with three sets of 5 words each learnt over three consecutive days and each word was practiced across three exercises within a day (i.e., three exposures). Over the same three-day period, the second list was exposed in DP fashion. Here, the list was learnt by students doing 1 exercise on each of the 15 words each day over three days (i.e., three exposures). However, there was a difference in retention interval between the final test and each day of the MP condition. As such, data is a comparison between a subset of words presented in MP fashion (i.e., those presented on Session 4 / Practice Session 3) and all words in the DP condition. | | Not reported | 3 | Fixed (1 day) | 35 | Language | Researcher | Retention - mean percentage of words correctly recalled | 46.06 (29.78) | 33 | 55.96 (26.24) | 33 | 0.35 | -0.13 | 0.84 | 0.25 |
| Greving & Richter | 2019 | Germany | Published Empirical Study | Between | 62 | Across all conditions - 12.94 (0.39) | Secondary | MP participants read a 977 word text about a biology concept twice and then completed filler tasks. They were tested on recall and text comprehension 1 weeks later | DP participants read the same text and then completed filler tasks for the remainder of the session. A week later, they read the text again and completed additional filler tasks and activities. After another week, participants were tested on recall and text comprehension. | Not reported | 1 | Fixed (7 days) | 7 | Science | Researcher | Retention - Mean number of ideas remember in a 2-minute free recall session (out of 5) | 1.79 (2.04) | 36 | 2.34 (2.0396) | 26 | 0.27 | -0.24 | 0.78 | 0.26 |
| Grote | 1995 | USA | Published Empirical Study | Within | 36 | Not reported (US Grades 11-12) | Secondary | All participants learned two physics topics over two days and were then randomly assigned to a practice condition order. On the third and fourth day, participants were given 60 problems on one topic to complete (MP condition). For the next 20 days (Days 4-24), students received 3 problems per day on the second topic (DP condition). Testing occurred 2, 4 and 6 weeks after the final DP practice session. | | Not reported | 3 | Fixed (1 day) | 42 | Science | Researcher | Retention - mean number of multiple choice questions answered correctly on each topic (each out of 20) | Not reported | 36 | Not reported | 36 | 0.57 | NA | NA | NA |
| * Holdan | 1986 | USA | Unpublished Dissertation | Between | 28 | Not reported (US Grade 8) | Secondary | Students in the 'Traditional' condition practiced all items related to one of the three lessons on each of two topics the day after that lesson was presented in class. | Students in the 'Distributed' condition completed some of the problems from the first lesson after it was presented in class, with the remaining problems interspersed in the practice of following lessons (i.e., some problems from lesson 1 and lesson two were practiced after lesson 2 was studied, with the remaining problems from lesson 1 and lesson 2 were practiced alongside all problems from lesson 3 after lesson 3 was studied) | Not reported | Unclear | Fixed (7 days) | Unspecified | Maths | Researcher | Retention - mean t-score on test of two problem types | 46.9 (10.0) | 14 | 50.6 (9.8) | 14 | 0.37 | -0.37 | 1.12 | 0.38 |

| | | | | | | | | | | | | | | | | | | | | | | | | |
|---|---|---|---|---|---|---|---|---|---|---|---|---|---|---|---|---|---|---|---|---|---|---|---|---|
| Küpper-Tetzel, Erdfelder & Dickhäuser | 2014(a) - 1-day lag | | | | 22 | | | | | | 1 | Fixed (1 day) | | | | Retention - mean percentage of words recalled | 42.2 (18.71) | 12 | 58.2 (12.97) | 10 | 0.98 | 0.09 | 1.86 | 0.45 |
| Küpper-Tetzel, Erdfelder & Dickhäuser | 2014(b) - 7-day lag | Germany | Published Empirical Study | Between | 20 | 11.45 (SD not reported) | Secondary | In the initial session, 26 German-English pairs were presented within two study-test trials took place (each involving presentation of the word pairs, followed by a recognition test, a cued recall test and a picture quiz test). The MP condition received a third study-test trial immediately after this. | The DP condition had a lag of either 1 or 10 days before the third study-test trial | Approx. 90 minutes | 1 | Fixed (10 days) | 35 | Language | Researcher | Retention - mean percentage of words recalled | 42.2 (18.71) | 12 | 54.3 (19.52) | 8 | 0.64 | -0.28 | 1.55 | 0.47 |
| ^ Küpper-Tetzel, Erdfelder & Dickhäuser | 2014 - Average (across two lag conditions) | | | | | | | | | | 1 | | | | | Retention - combined mean for (1) and (2) above | 42.2 (18.2987) | 24 | 56.4667 (15.8092) | 18 | 0.83 | 0.19 | 1.46 | 0.32 |
| Lotfolahi & Salehi | 2017 | Iran | Published Empirical Study | Within | 28 | 9.17 (NA) | Primary | Two lists of 10 word pairs (counterbalanced across two classes) were randomly selected from a sample of 20 English-Farsi word pairs. Within the MP condition, Day 1 saw students learn 5 word pairs from one list by drilled practice in pairs with corrective feedback, choral repetition and another round of drilled feedback. On Day 2, the MP condition involved the same learning session with the other 5 words from the list. In the DP condition, participants learnt the full 10 word pairs on Day 1 using the same learning approach and then again on Day 2. | | Approx. 40 minutes | 1 | Fixed (1 day) | 35 | Language | Researcher | Mean percentage of correct recall | 28.57 (12.0646) | 28 | 50.71 (15.3454) | 28 | 1.60 | 1.00 | 2.21 | 0.31 |
| * Lyle, Bego, Ralston & Immekus | 2022 | USA | Published Empirical Study | Within | 180 | Not reported (University undergraduate) | University | 24 learning objectives were practice using three questions. Objectives presented under MP conditions were practice using three variations of the question for that objective given within the same quiz. This quiz was given the same week as the last of the 8 objectives captured in the preceding three weeks was covered. Objectives presented under DP conditions were practiced by participants completing 1 variant of the question in each of 3 weekly quizzes. The first weekly quiz was given after the last of the 8 objectives was presented in class, with the remaining two quizzes taking place weekly after this. | | 3 minutes per quiz question | 3 | Fixed (7 days) | Unequal | Maths | Teacher | Mean proportion correct on criterial test (out of 1) | 0.71 (0.18) | 180 | 0.77 (0.17) | 180 | 0.34 | 0.13 | 0.55 | 0.11 |
| McNellis | 2015 | USA | Published Empirical Study | Between | 69 | Not reported (University undergraduate) | University | MP participants received a 120-minute session (spaced over two classes) on understanding and preparing a Statement of Cash Flows (SCF). This occurred in the last week of the semester. For each exercise/problem reviewed from earlier in the semester, the lecturer explored how they connect to the rules associated with the SCF preparation. | Students in the DP condition received information on SCF preparation in the context of each of the 12 chapters covered weekly, with approximately 10 minutes devoted to this in each class (i.e., 120 minutes) | 120 minutes | 1 | Fixed (7 days) | 7 | Accounting | Teacher | Mean percentage of SCF material incorporated into SCF preparation task on the final exam (out of 34 items) | 0.464 (0.191) | 30 | 0.594 (0.174) | 39 | 0.67 | 0.18 | 1.16 | 0.25 |

| | | | | | | | | | | | | | | | | | | | | | | | | |
|---|---|---|---|---|---|---|---|---|---|---|---|---|---|---|---|---|---|---|---|---|---|---|---|---|
| Moss | 1996 (1) - Grade 2 Reading | USA | Unpublished Dissertation | Between | 85 | Not reported (US Grade 2 & 4) | Primary | 30-minutes of instruction on reading on Day 1 and 30 minutes of instruction on Maths on Day 2, each week for 9 weeks | 15 minutes of maths and 15 minutes of English (back-to-back) on Day 1 and again on Day 2, each for 9 weeks. | 270 minutes per study / skill area | 18 | Fixed (1 day) | Immediately after the treatment at the next assigned computer class time | Reading and Maths | Teacher | Percentage correct on final test | 67.19 (22.09) | 42 | 64.81 (20.37) | 43 | -0.11 | -0.54 | 0.31 | 0.22 |
| Moss | 1996 (2) - Grade 2 Maths | | | | 120 | | | | | | 18 | | | | | | 74.19 (16.12) | 60 | 71.02 (17.70) | 60 | -0.19 | -0.55 | 0.17 | 0.18 |
| Moss | 1996 (3) - Grade 4 Reading | | | | 74 | | | | | | 18 | | | | | | 90.97 (13.16) | 31 | 89.72 (12.08) | 43 | -0.10 | -0.56 | 0.36 | 0.24 |
| Moss | 1996 (4) - Grade 4 Maths | | | | 120 | | | | | | 18 | | | | | | 50.87 (14.68) | 60 | 51.41 (12.72) | 60 | 0.04 | -0.32 | 0.40 | 0.18 |
| ^ Moss | 1996 (5) - Grade 2 Average (across reading & maths) | | | | | | | | | | 18 | | | | | | 71.3076 (19.0228) | 102 | 68.4275 (19.0143) | 103 | -0.15 | -0.43 | 0.12 | 0.14 |
| ^ Moss | 1996 (6) - Grade 4 Average (across reading & maths) | | | | | | | | | | 18 | | | | | | 64.5304 (23.7527) | 91 | 67.4035 (22.6737) | 103 | 0.12 | -0.16 | 0.41 | 0.14 |
| ^ Moss | 1996 (7) - Average (across age and learning domain) | | | | | | | | | | 18 | | | | | | 68.1122 (21.5946) | 193 | 67.9155 (20.8793) | 206 | -0.01 | -0.21 | 0.19 | 0.10 |
| Nazari & Ebersbach | 2018 | Germany | Published Empirical Study | Between | 44 | 16 years, 6 months | Secondary | Following three lectures, participants in the MP condition received three practice sets (each consisting of 4 practice problems) for the studied content the day after the last lecture. | In the DP condition, participants received the same three practice sets - one the day after the last practice session, one two days after this and then the third 5 days after that. | Not reported (students had a maximum 36 hours to complete each set) | 3 | Expanding (1, 3 and 8 days after initial exposure) | 14 | Maths | Researcher | Mean score on test session (out of 15) | | 27 | | 27 | NA | NA | NA | NA |
| Nazari & Ebersbach | 2019(a) | Germany | Published Empirical Study | Between | 81 | 13.2 (NA) | Secondary | Following an introductory 90-minute lecture on the content, participants in the MP condition completed three practice sets involving three problems about the content. Filler activities were given during the time the DP condition were re-exposed to the material | In the DP condition, participants received the same three practice sets - one 5-6 days after the last practice session, one two days after this and then the third 5 days after that. | 45 minutes | 3 | Expanding (2 days between 1st and 2nd practice, then 5 between 2nd and 3rd) | 42 | Maths | Researcher | Mean score on post-test (out of 9.5) | 5.90 (3.03) | 36 | 6.87 (2.81) | 45 | 0.33 | -0.11 | 0.77 | 0.23 |

| | | | | | | | | | | | | | | | | | | | | | | | | |
|---|---|---|---|---|---|---|---|---|---|---|---|---|---|---|---|---|---|---|---|---|---|---|---|---|
| Nazari & Ebersbach | 2019(b) (1) Grade 3 students, semi-formal multiplication method | Germany | Published Empirical Study | Between | 95 | 9.5 (NA) | Primary & Secondary | One day after an introductory 90-minute session presenting the content, participants in the MP condition completed three practice sets each containing four problems related to the content. Students were given 10 minutes to complete each practice set. | DP condition participants completed one practice set each day for three days. | 30 minutes | 3 | Fixed (1 day) | 42 | Maths | Researcher | Mean percentage on post-test (out of 12) | 86% (22.87147 %) | 50 | 92% (16.64263 %) | 45 | 0.30 | -0.11 | 0.70 | 0.21 |
| Nazari & Ebersbach | 2019(b) (2) Grade 7 students, stochastics | | | | 118 | 13 years, 5 months | | Five to seven days after 180 minutes of introductory content (spread over two 90-minute sessions each day over two days), students in the MP condition were given three practice sets consisting of three problems each. The maximum time given for students to complete each one was 15 minutes | DP condition participants completed one practice set each day for three days. | 45 minutes | 3 | | | | | | 45% (29.28408 %) | 61 | 51% (32.03489 %) | 57 | 0.20 | -0.17 | 0.56 | 0.18 |
| Petersen-Brown, Lundberg, Ray, Dela Paz, Riss & Panahon | 2019 | USA | Published Empirical Study | Between | 61 | Not reported (US Grad 3 and 4) | Primary | Following a 15-20 minute teaching session, students immediately completed 3 practice sessions where they practiced words until they correctly recalled and defined 100% of the 8 words. | Participants in the DP condition completed the same 3 practice sessions recalling words to a criterion of perfect performance, with sessions spaced over a number of days according to either a fixed or expanding interstudy interval | Not reported (students were trained to a criterion of perfect performance within conditions) | 3 | Fixed (7 days) and Expanding (7, then 12) | 7 | Maths | Researcher | Mean number of words recalled (out of 8) | 4.36 (1.94) (22) | 22 | 5.56 (1.86) (39) | 39 | 0.64 | 0.10 | 1.17 | 0.27 |
| Petersen-Brown, Riese, Schneider, Ray & Clonkey | 2023 | USA | Published Empirical Study | Between | 88 | Not reported (US Grades 2 and 3) | Primary | MP-condition participants completed an initial teaching session followed by 20-minutes of practice in which they were exposed to each of the 8 words 8 times across 8 pages of the practice packet | DP-condition participants had two pages to practice two words (in either a massed or distributed within-session spacing condition) in each of four sessions. The sessions were spread approximately 1 week apart | 20 minutes (or 8 practice opportunities per word, whichever was shorter) | 3 | Fixed (7 days) | 28.3 | Spelling | Researcher | Mean number of correctly spelled words (out of | Not reported (F-test results used) | 44 | Not reported (F-test results used) | 44 | 0.63 | 0.03 | 1.24 | 0.31 |
| Reynolds & Glaser | 1964 | USA | Published Empirical Study | Between | 58 | Not reported (US Junior High School) | Secondary | A Grolier Min-Max II teaching machine (presenting information via sequentially presented frames or slides) was used to expose participants in the MP condition to 11 new words related to a science concept (i.e., Mitosis). The words were exposed a fixed number of times across one or two 40-minute classes. | The same platform was used to expose participants in the DP condition to the same material. These participants had 2/3 the exposures in an initial session, with the remaining 1/3 split over two additional sessions; the first occurred approximately two days after the initial session, with the second being held 2 days after this. | Not reported (number of within-session repetition held constant across conditions) | 2 | Fixed (1-2 days) | 31 | Science | Teacher | Mean number of words correctly remembered via an unaided recall test (out of 22, i.e., two questions about each word) | 7.00 (3.49) | 35 | 11.74 (4.21) | 23 | 1.25 | 0.68 | 1.82 | 0.29 |

| | | | | | | | | | | | | | | | | | | | | | | | | |
|---|---|---|---|---|---|---|---|---|---|---|---|---|---|---|---|---|---|---|---|---|---|---|---|---|
| Sayeski, Earle, Eslinger & Whitenton | 2017 | USA | Published Empirical Study | Between | 52 | Not reported | University | All participants received a 1.5 initial learning 'interactive lecture' in which they were exposed to general concepts related to the alphabetic principles and the teaching strategy of Constant Time Delay (CTD). Participants in the MP condition then engaged in a 60-minute practice session in which they worked in pairs and used digitial flashcards and audio recordings to practice phoneme-grapheme pairs. | Following the same initial learning session, participants in the DP class received a 15-minute practice window at the beginning of each weekly class over the next four weeks. | 60 minutes | 4 | Fixed (7 days) | 28 | Teacher Education - Phoneme/Grapheme | Teacher | Mean number of phonemes correctly produced (out of 75) | 47.67 (8.94) | 26 | 60.67 (4.95) | 26 | 1.80 | 1.15 | 2.44 | 0.33 |
| * Weaver | 1976 (1) - Percent | | | | | | | | | | Unclear | | | | | | 5.34 (2.81) | 147 | 5.21 (2.84) | 143 | -0.05 | -0.28 | 0.18 | 0.12 |
| * Weaver | 1976 (2) - Real Number | USA | Unpublished Dissertation | Between | 293 | Not reported (US Grade 8) | Secondary | Participants in the MP condition were assigned all problems for concepts in each area of study (percentages and real numbers) the day after the content for that concept was introduced. | Participants in the DP condition was assigned the same questions for each content, but the problems were distributed across multiple homework tasks. The number of problems assigned was based on a formula, with the formula using the number of days after the content was introduced to calculate the number of problems from one concept to be assigned. | Not reported, assumed equal (number of problems was constant across conditions) | Unclear | Expanding (1, 2, 4, 7, 12, 19, 29) | Unspecified | Maths | Teacher | Mean number of items correctly items (out of 12) | 4.19 (2.31) | 147 | 4.28 (2.36) | 143 | 0.04 | -0.19 | 0.27 | 0.12 |
| * Weaver | 1976 (3) - Average (across content areas in (1) and (2)) | | | | | | | | | | Unclear | | | | | | 4.765 (2.6315) | 294 | 4.745 (2.6478) | 286 | -0.01 | -0.17 | 0.16 | 0.08 |
| * Yazdani & Zebrowski | 2006 | USA | Published Empirical Study | Between | 120 | Not reported (US Grade 10 and 11) | Secondary | MP condition participants were exposed to content (including the focus topic) and then provided homework that covered one area of content only | In the DP condition, participants were presented with content in the same way as the MP condition. Homework relating to the focus topic was distributed over a number of weeks, with each homework assignment consisting of a proportion of questions relating to that topic | Not reported (authors noted "virtually identical" (p. 39) | Unclear | Variable (1-6 days) | Unspecified | Maths | Teacher | Mean score on van Hiele Geometry Test | 19.63 (1.52) | 61 | 24.25 (1.45) | 59 | 3.11 | 2.58 | 3.64 | 0.27 |

*Notes*.

* denotes studies with unequal / unspecified retention intervals.

^ denotes effect sizes that are calculated as the combined mean and standard deviation of the relevant experiments/groups.

[#] denotes arbitrary numbering of the multiple effect sizes presented in a single study.

**R-Code for Meta-Analysis**

```
> library(metafor)
> data.frame(MA_1_k_22_All_studies_all_effects$`Study Label`,MA_1_k_22_All_studies_all_effects$SMD,MA_1_k_22_All_studies_all_effects$SE)
            MA_1_k_22_All_studies_all_effects..Study.Label.
1                                  Bloom & Shuell 1981
2                                     * Buzzelli 2014
3                        * Camp 1973 (1) - Polynomials
4              * Camp 1973 (2) - Linear Equations & Graphs
5           Emeny, Hartwig & Rohrer 2021 (1) - Experiment 1
6           Emeny, Hartwig & Rohrer 2021 (2) - Experiment 2
7                Foot-Seymour, Foot & Wiseheart 2019 (3)
8                Foot-Seymour, Foot & Wiseheart 2019 (4)
9                    Foot-Seymour & Wiseheart 2022 (3)
10                                Goossens et al. 2012
11                               Greving & Richter 2019
12                                      * Holdan 1986
13 Küpper-tetzel, Erdfelder & Dickhäuser 2014 (1) - 1-day lag
14 Küpper-tetzel, Erdfelder & Dickhäuser 2014 (2) - 7-day lag
15                               Lotfolahi & Salehi  2017
16                                  * Lyle et al. 2022
17                                      McNellis 2015
18                         Moss 1996 (1) - Grade 2 Reading
19                          Moss 1996 (2) - Grade 2 Maths
20                         Moss 1996 (3) - Grade 4 Reading
21                          Moss 1996 (4) - Grade 4 Maths
22                            Nazari & Ebersbach 2019(a)
23          Nazari & Ebersbach 2019(b) (1) - Grade 3 students
24          Nazari & Ebersbach 2019(b) (2) - Grade 7 students
25                            Petersen-Brown et al. 2019
26                            Petersen-Brown et al. 2023
```

| | |
|---|---|
| 27 | Reynolds & Glaser 1964 |
| 28 | Sayeski et al. 2017 |
| 29 | * Weaver 1976 (1) - Percent |
| 30 | * Weaver 1976 (2) - Real Number |
| 31 | * Yazdani & Zebrowski 2006 |

| | MA_1_k_22_All_studies_all_effects.SMD | MA_1_k_22_All_studies_all_effects.SE |
|---|---|---|
| 1 | 0.9970 | 0.29405612 |
| 2 | 0.2181 | 0.44852041 |
| 3 | 0.4410 | 0.15854592 |
| 4 | 0.2845 | 0.15742347 |
| 5 | 0.7433 | 0.22043367 |
| 6 | 0.5384 | 0.19410714 |
| 7 | 0.8398 | 0.10969388 |
| 8 | 0.1982 | 0.10525510 |
| 9 | 0.2071 | 0.07497449 |
| 10 | 0.3527 | 0.24808674 |
| 11 | 0.2696 | 0.25849490 |
| 12 | 0.3737 | 0.38125000 |
| 13 | 0.9769 | 0.45278061 |
| 14 | 0.6359 | 0.46737245 |
| 15 | 1.6040 | 0.30724490 |
| 16 | 0.3427 | 0.10617347 |
| 17 | 0.6692 | 0.24943878 |
| 18 | -0.1121 | 0.21711735 |
| 19 | -0.1873 | 0.18298469 |
| 20 | -0.0997 | 0.23573980 |
| 21 | 0.0393 | 0.18260204 |
| 22 | 0.3334 | 0.22512755 |
| 23 | 0.2975 | 0.20660714 |
| 24 | 0.1958 | 0.18464286 |
| 25 | 0.6353 | 0.27275510 |
| 26 | 0.6346 | 0.30900510 |

| | | |
|---|---|---|
| 27 | 1.2509 | 0.29247449 |
| 28 | 1.7991 | 0.32869898 |
| 29 | -0.0460 | 0.11747449 |
| 30 | 0.0385 | 0.11747449 |
| 31 | 3.1090 | 0.27132653 |

```
> rma(MA_1_k_22_All_studies_all_effects$SMD,sei=MA_1_k_22_All_studies_all_effects$SE)
```
